# Supplementary material for: Double-pentagon silicon chains in a quasi-1D Si/Ag(001) surface alloy
Source: Nat Commun. 2024 Oct 25;15:9242. doi: 10.1038/s41467-024-53589-4 (PMC11511886; doi:10.1038/s41467-024-53589-4)
Supplement: Supplementary file 1 — Supplementary Information [file 41467_2024_53589_MOESM1_ESM.pdf]

SUPPLEMENTARY INFORMATION

Double-pentagon silicon chains in a quasi-1D Si/Ag(001) surface alloy

Conor Hogan,[1, 2, *] Andrea Sette,[2] Vasil A. Saroka,[2] Stefano Colonna,[1] Roberto Flammini,[1] Laurita Florean,[3, 4] Romain Bernard,[3] Laurence Masson,[5] Geoffroy Prévot,[3] and Fabio Ronci[1, †]

[1] *CNR-Istituto di Struttura della Materia (CNR-ISM), Via Fosso del Cavaliere 100, 00133 Rome, Italy*

[2] *Dipartimento di Fisica, Università di Roma "Tor Vergata", Via della Ricerca Scientifica 1, 00133 Rome, Italy*

[3] *Sorbonne Université, CNRS-UMR 7588, Institut des NanoSciences de Paris, F-75005, Paris, France*

[4] *Laboratoire de Chimie Physique Matière et Rayonnement, UMR7614, Sorbonne Université, CNRS, Paris F-75005, France*

[5] *Aix Marseille Univ, CNRS, CINaM, AMUtech, Marseille, France*

LIST OF FIGURES

1 LEED patterns 2
2 STM image of a low coverage area 3
3 Full set of bulk truncated models 4
4 Full set of surface alloy models 5
5 Phase diagram for full set of models 6
6 PDOS calculations 7
7 Bias dependent STM images 8
8 SXRD fits for full set of models 9

* conor.hogan@cnr.it

† fabio.ronci@cnr.it

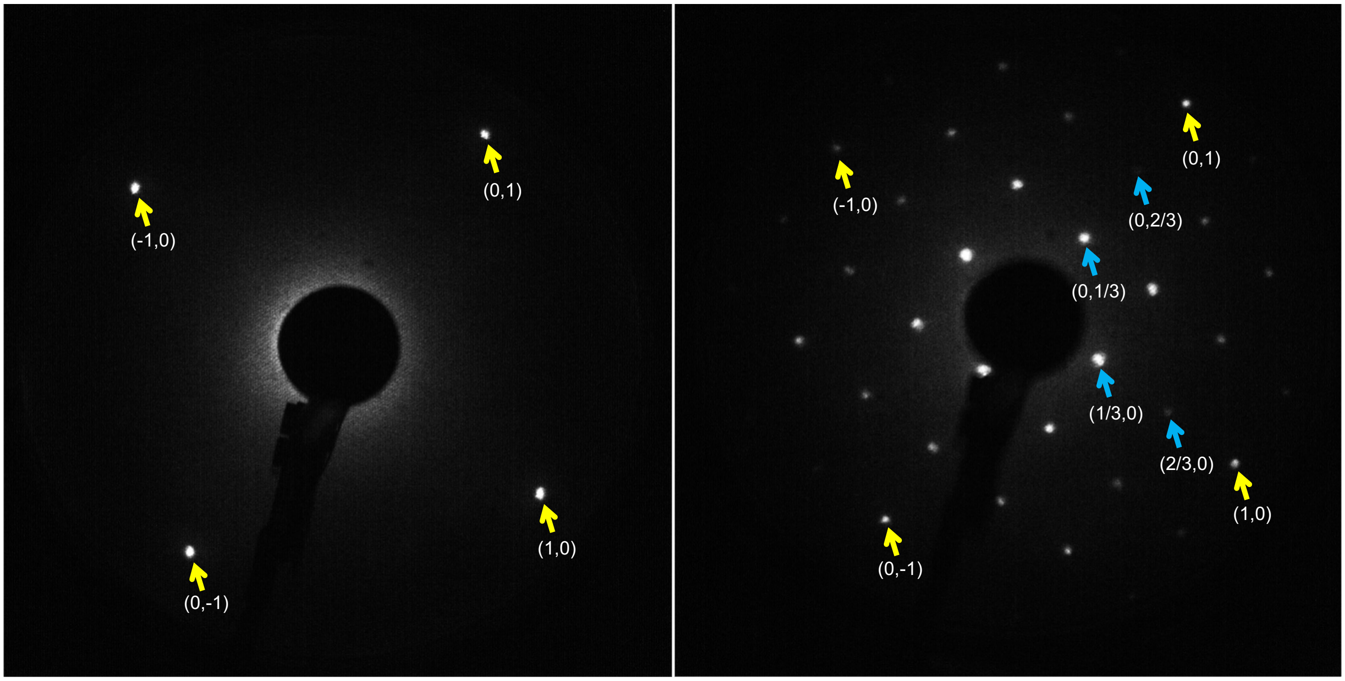

Supplementary Figure 1. **LEED patterns before and after Si deposition.** LEED patterns ($E = 40$ eV) of the clean Ag(001) substrate before (left panel, 1×1) and after (right panel, 3×3) 0.8 ML Si deposition with the sample held at 490 K. Yellow arrows indicate the integer order spots; blue arrows the fractional order spots along the $+X$ and $+Y$ directions.

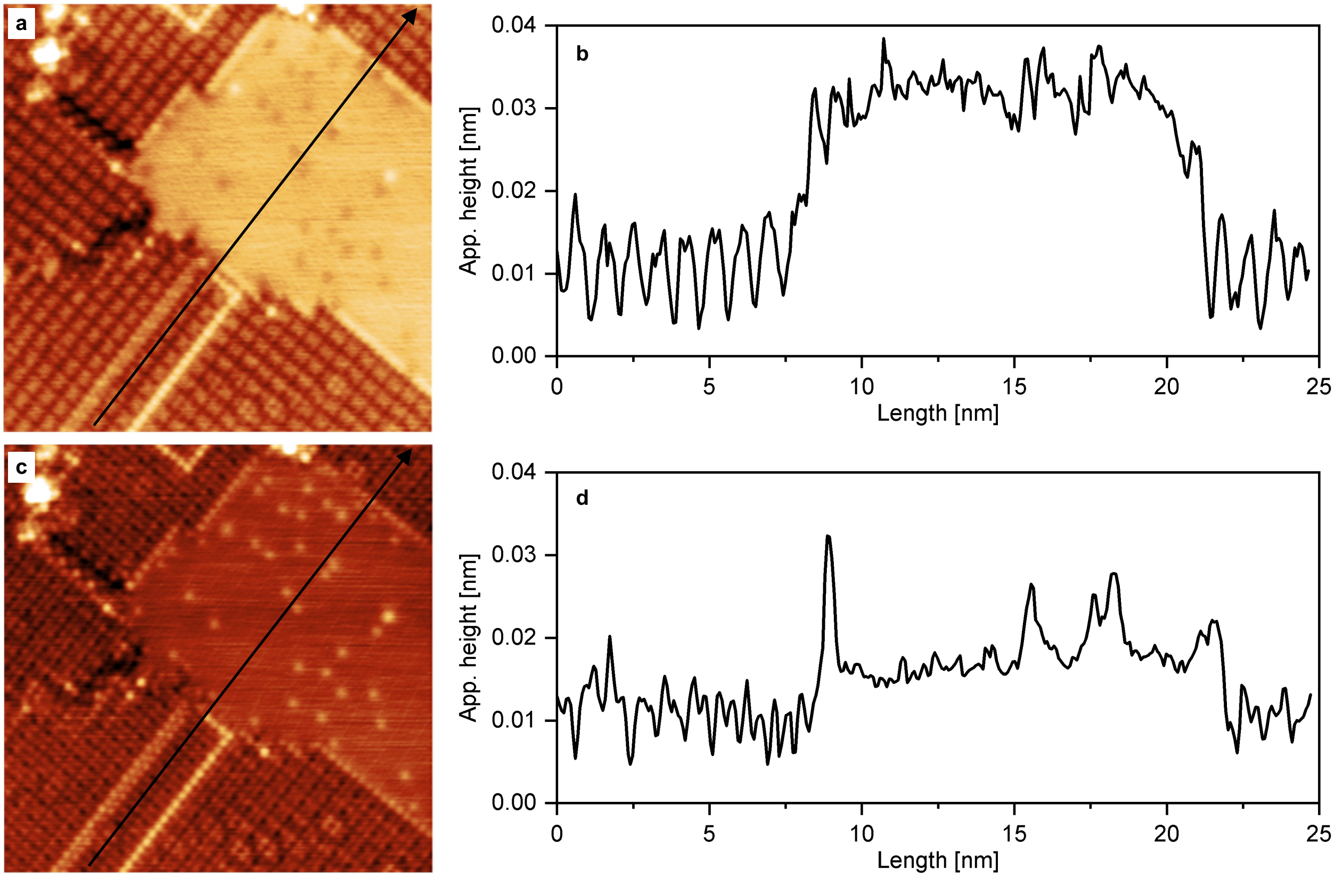

Supplementary Figure 2. **STM image of a low coverage area.** **a** Empty state STM image (20 × 20 nm^2, V_s = +1 V, I = 2 nA) showing a Ag(001) terrace adjacent to 3 × 3 domains. **b** Line profile along the black arrow shown in **a**. **c,d** Filled state STM image and line profile (V_s = -1 V, I = 2 nA). Source data are provided as a Source Data file.

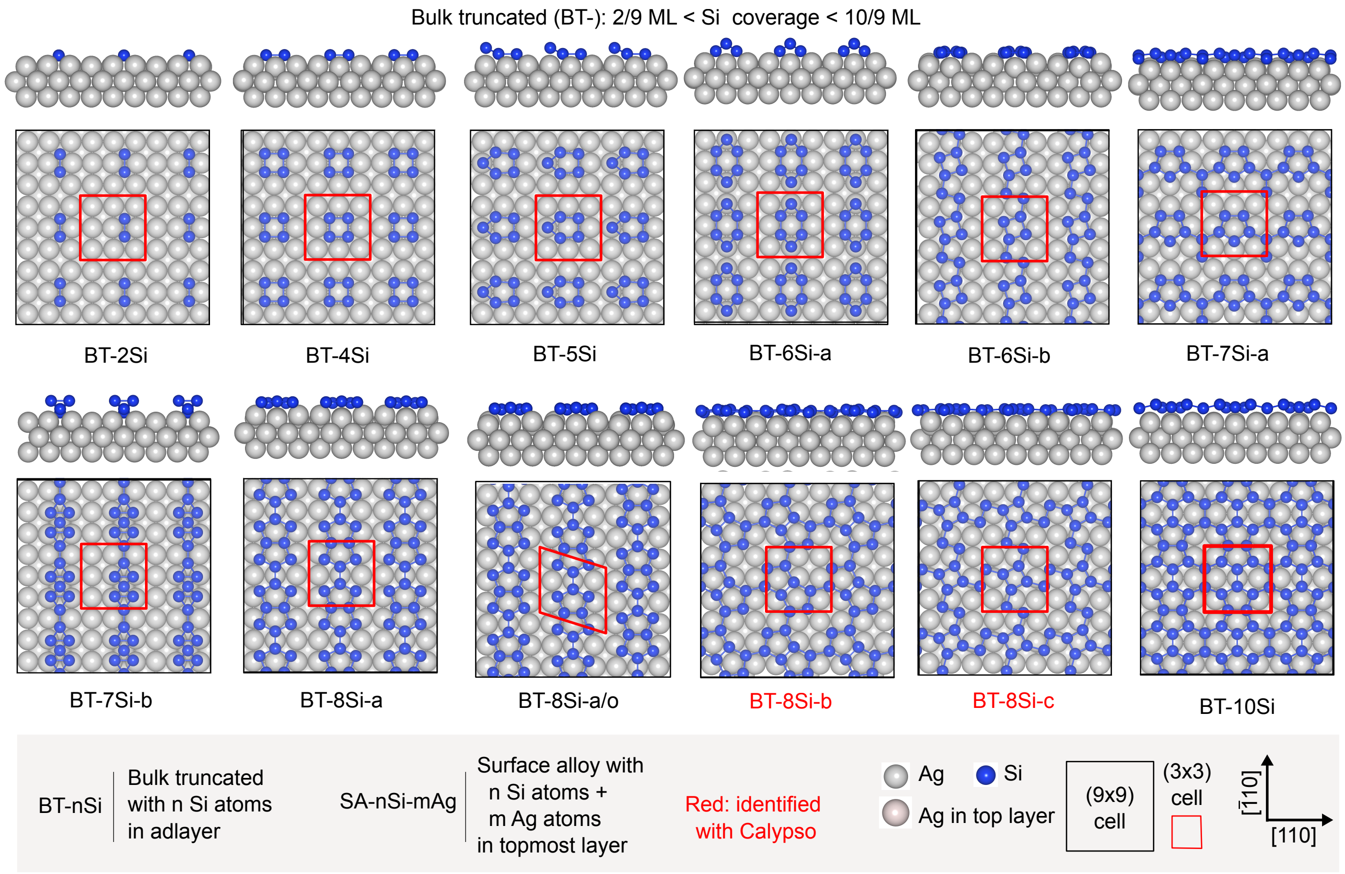

Supplementary Figure 3. **Bulk truncated structural models.** Full set of optimized bulk truncated schematic models of the Si/Ag(001)-(3 × 3) surface, side and top views. Topmost three or four layers are shown. Unit cells are indicated by red squares/rhomboids.

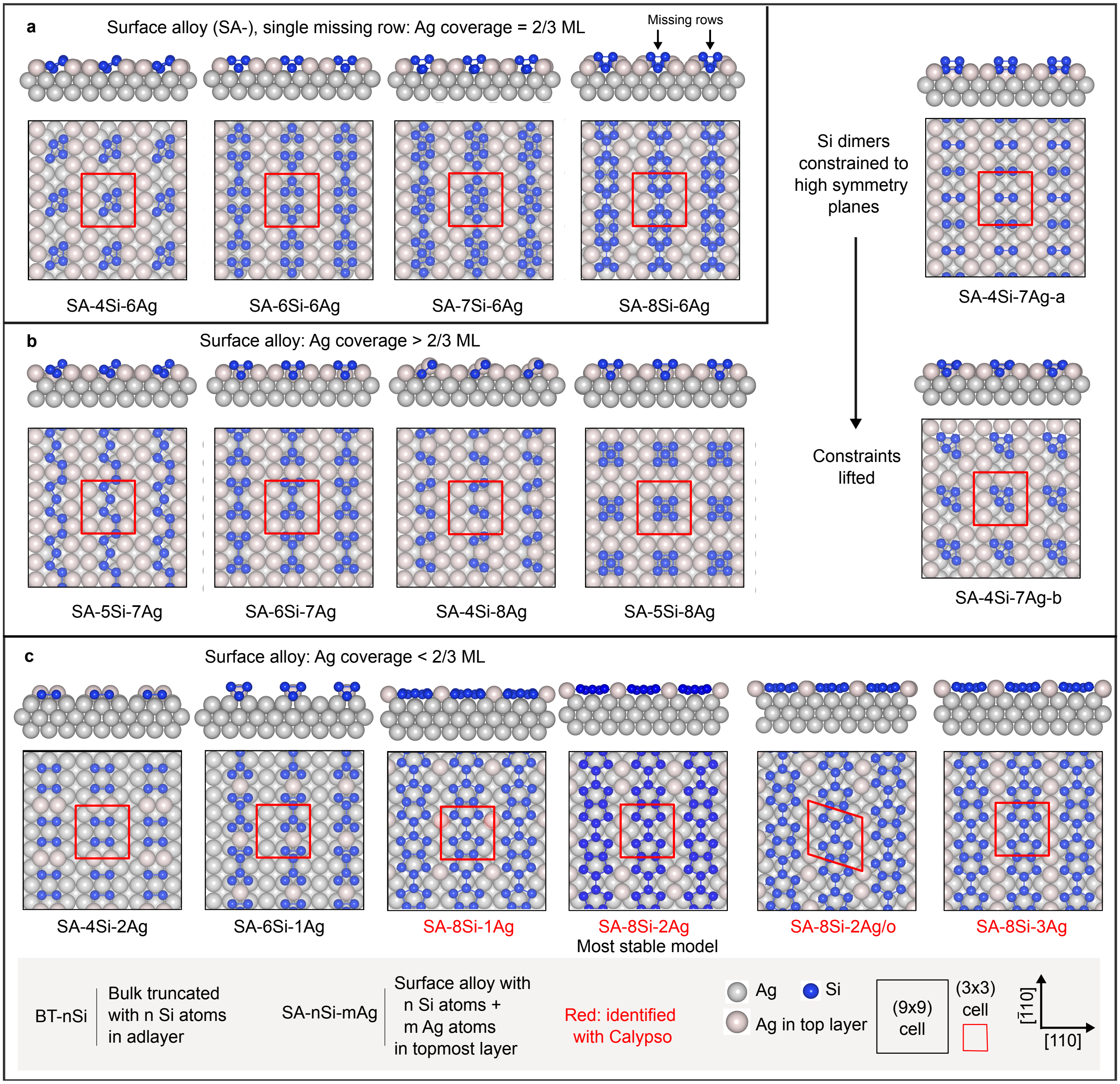

Supplementary Figure 4. **Surface alloy structural models.** Full set of optimized surface alloy schematic models of the Si/Ag(001)-(3×3) surface, side and top views. Topmost three or four layers are shown. **a** Models based on a single Ag missing row substrate geometry, i.e. 2/3 ML Ag coverage. **b** Surface alloy models having Ag coverage above 2/3 ML. **c** Surface alloy models having Ag coverage below 2/3 ML. The most stable model, SA-8Si-2Ag, is also referred to as SA-DPC. Unit cells are indicated by red squares/rhomboids.

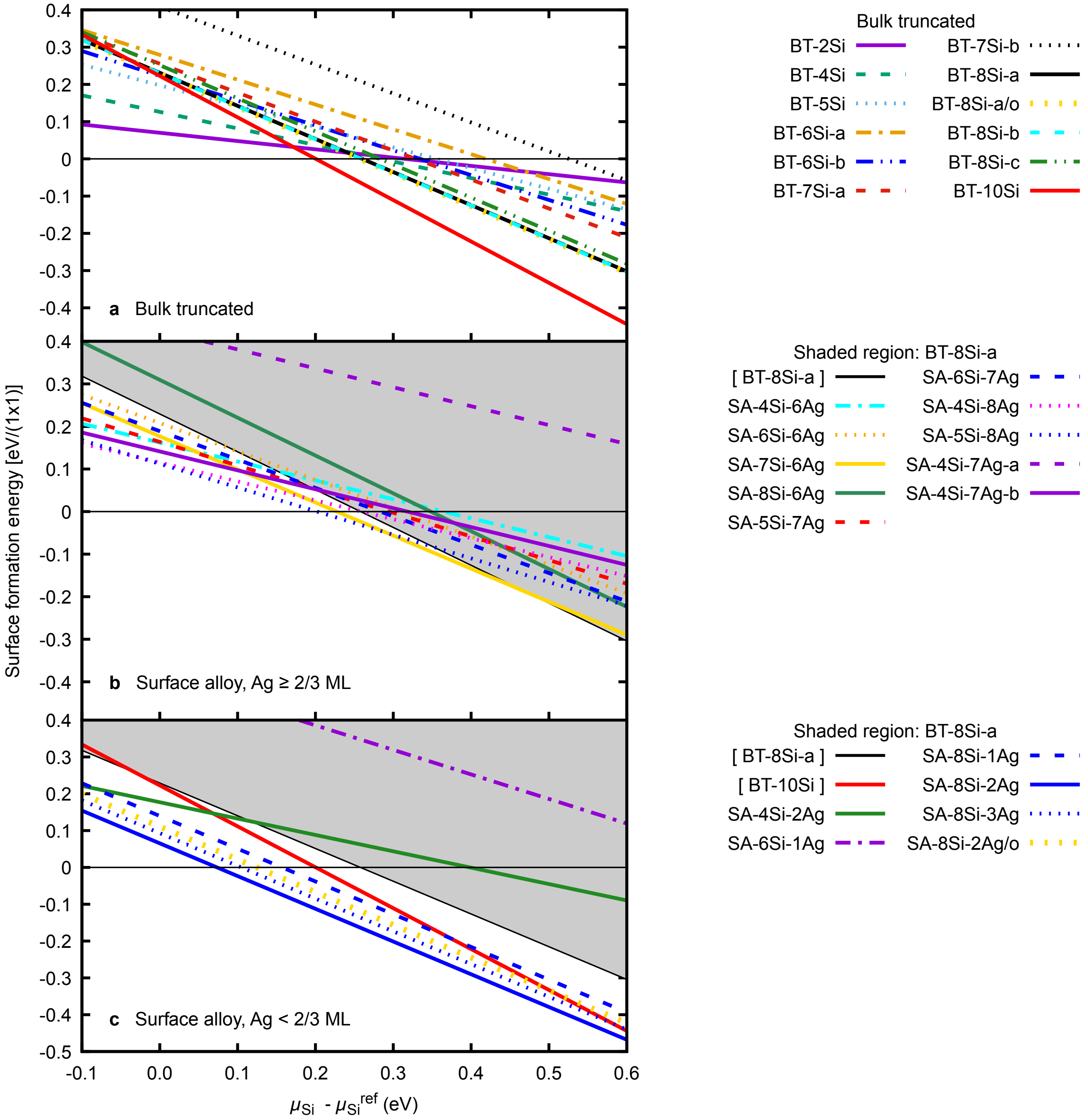

Supplementary Figure 5. **Phase diagram for full set of models.** Surface formation energies of Si/Ag(001)-(3 × 3) models, relative to that of the clean Ag(001) surface. **a** Bulk-truncated models, as presented in Supplementary Fig. 3. **b** Surface-alloy models, for higher Ag coverage, as presented in Supplementary Fig. 4a,b. The shaded area indicates the range of stability of the BT-8Si-a model. **c** Surface-alloy models, for lower Ag coverage, as presented in Supplementary Fig. 4c. The BT-10Si data is shown again for comparison. The most stable model is SA-8Si-2Ag (SA-DPC). Source data are provided as a Source Data file.

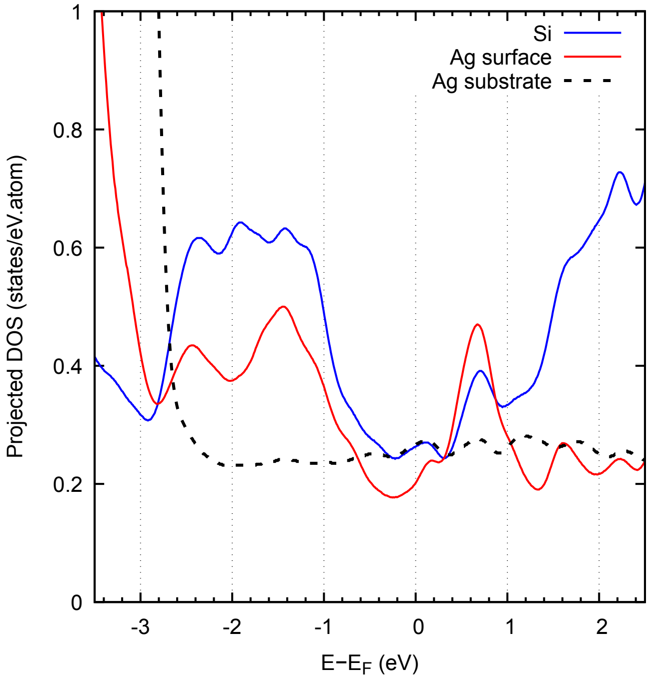

Supplementary Figure 6. **PDOS calculations.** Projected density of states calculation of the SA-DPC (SA-8Si-2Ag) model. The peak at +0.6 eV arising from surface Ag atoms is responsible for the apparent shift of the STM image for larger positive biases. Source data are provided as a Source Data file.

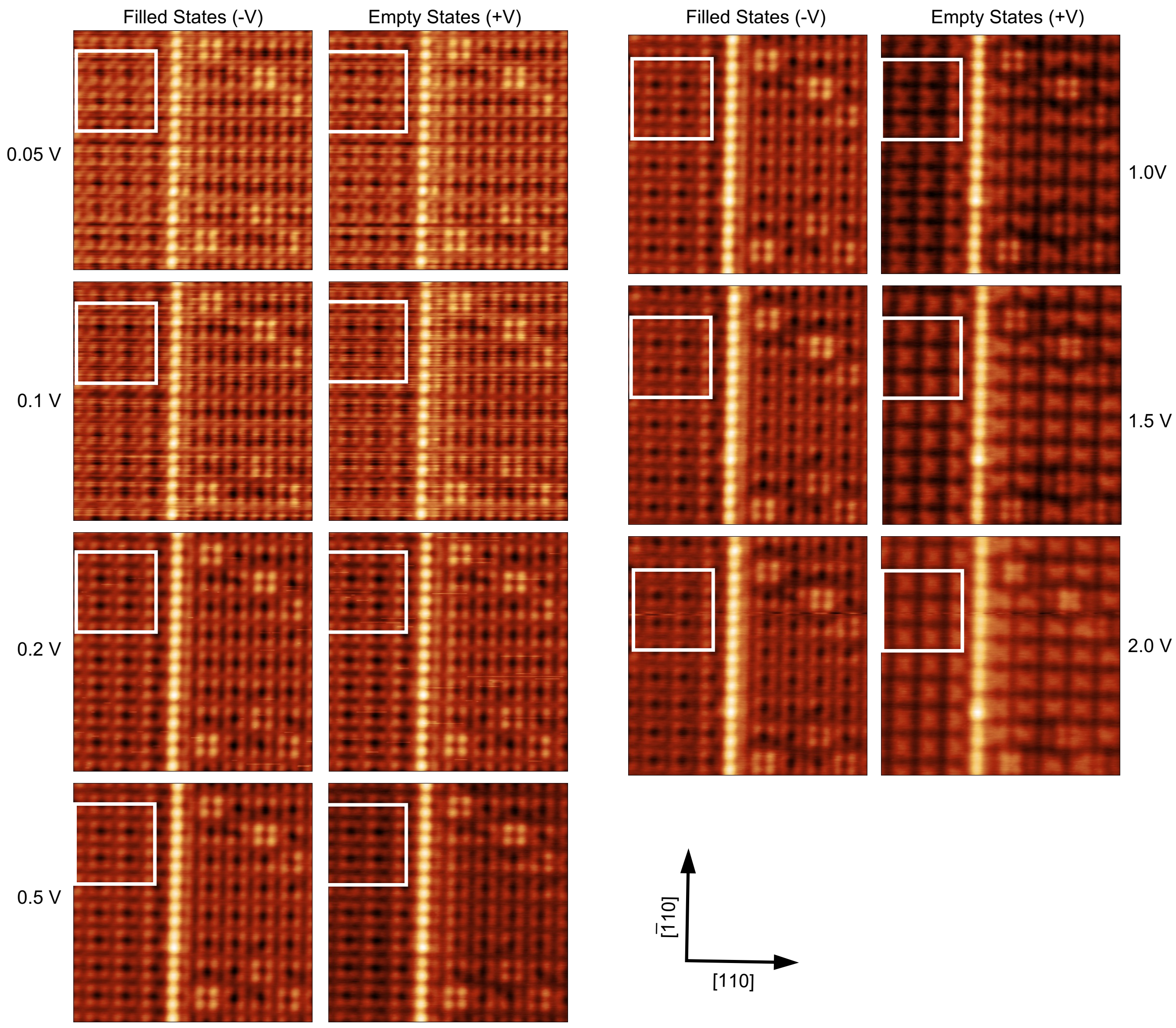

Supplementary Figure 7. **Large-scale high-resolution bias-dependent STM images**. Experimental 7.5×7.5 nm^2 STM images collected at 80 K with tunneling current I = 20 nA, at the bias indicated. White squares highlight the 2.6 nm areas reported in Fig. 3.

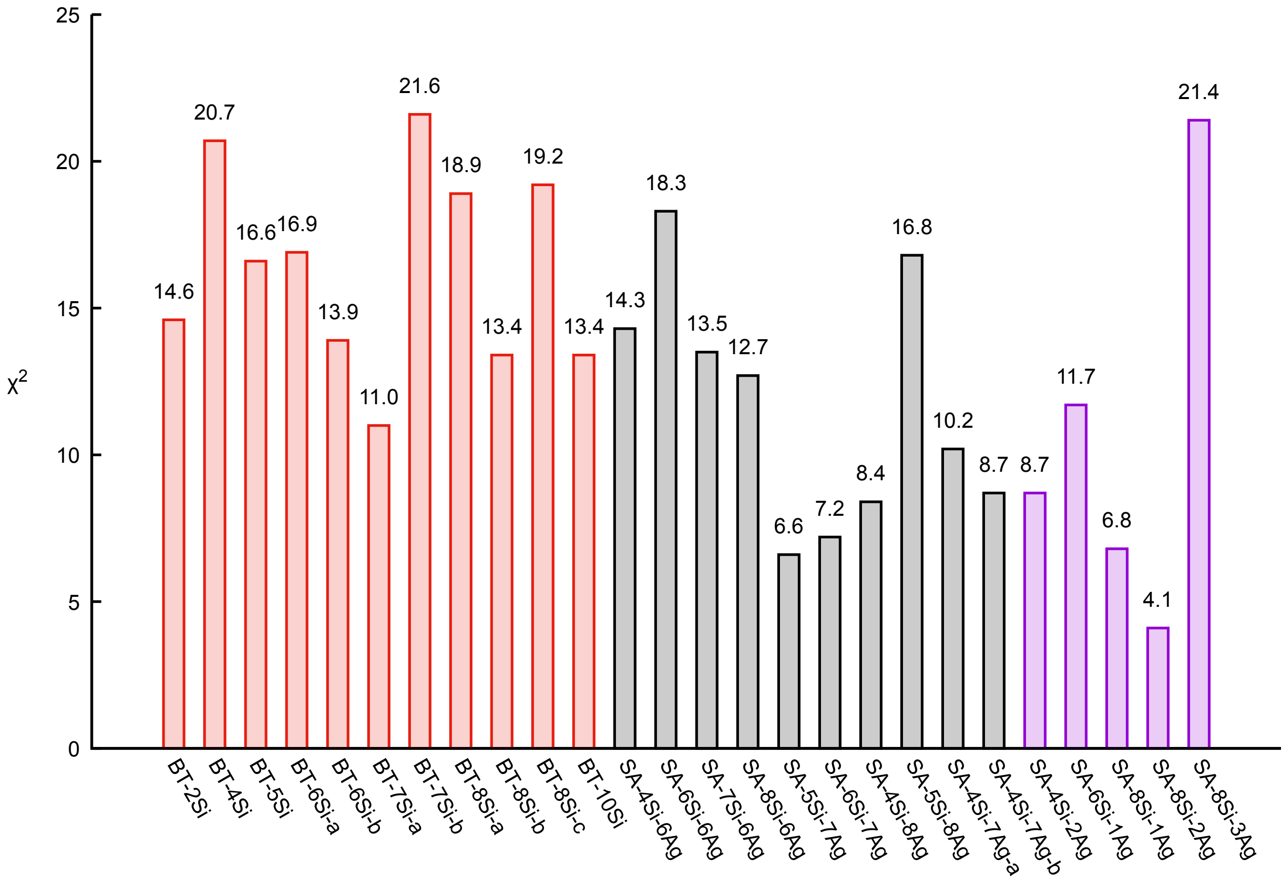

Supplementary Figure 8. **SXRD fits for full set of models.** Surface x-ray diffraction fits (χ^2) to experimental data in Leandri et al[1] for the Si/Ag(001)-(3×3) models shown in Supplementary Figs. 3 and 4. The best fit is found for the SA-8Si-2Ag (SA-DPC) model. Different shadings reflect the same groupings used in Supplementary Fig. 5.

[1] C. Léandri, H. Oughaddou, B. Aufray, J. M. Gay, G. Le Lay, A. Ranguis, and Y. Garreau, Growth of Si nanostructures on Ag(0 0 1), Surface Science **601**, 262 (2007).
