# Supplementary material for: Double-pentagon silicon chains in a quasi-1D Si/Ag(001) surface alloy
Source: Nat Commun. 2024 Oct 25;15:9242. doi: 10.1038/s41467-024-53589-4 (PMC11511886; doi:10.1038/s41467-024-53589-4)
Supplement: Supplementary file 2 — Description of Additional Supplementary Files [file 41467_2024_53589_MOESM2_ESM.pdf]

## Description of Additional Supplementary Files

File Name: Supplementary Data 1
Description: The zipfile contains all optimized atomic geometries in the form of input files for quantum-ESPRESSO. Each file contains the cartesian coordinates of the atomic positions and cell vectors. The data format is explained in the supplied README file.
